# Oral Manifestations of Cowden Syndrome in a Young Patient: A Case Report With Clinical, Histological, and Genetic Findings

**DOI:** 10.1155/crid/7101797

**Published:** 2026-10-18

**Authors:** Gian Marco Podda, Lucia Borghetti, Federica Rocchetti, Niccolò Di Giosaffatte, Irene Bottillo, Paola Grammatico, Cira Rosaria Tiziana Di Gioia, Umberto Romeo

**Affiliations:** ^1^ Department of Oral and Maxillofacial Sciences Sapienza University of Rome Rome Italy uniroma1.it; ^2^ Laboratory of Medical Genetics Experimental Medicine Department San Camillo-Forlanini Hospital Sapienza University Rome Italy uniroma1.it; ^3^ Department of Radiological Oncological and Anatomical Pathology Sciences Sapienza University of Rome Rome Italy uniroma1.it

**Keywords:** case report, Cowden syndrome, early diagnosis, hamartoma, oral manifestations, papillomatosis, PTEN

## Abstract

**Aims:**

This study is aimed at describing the oral manifestations and diagnostic pathway of Cowden syndrome in a young patient, highlighting the role of dental professionals in early detection of PTEN‐related disorders.

**Case Presentation:**

A 25‐year‐old woman presented with long‐standing, progressive multifocal oral papillomatous lesions since childhood. Clinical examination revealed macrocephaly and extensive papillomatosis involving the lips, tongue, palate, and buccal mucosa. Histopathological analysis demonstrated verrucous epithelial proliferation with parakeratosis, acanthosis, elongated rete ridges, and low‐grade dysplasia. Based on the clinical and histological findings, genetic testing was performed and identified a pathogenic PTEN variant (c.388C>T; p.Arg130Ter), confirming the diagnosis of Cowden syndrome. The patient was subsequently enrolled in a multidisciplinary surveillance program.

**Conclusion:**

Persistent multifocal oral papillomatosis associated with systemic findings should raise suspicion for Cowden syndrome. Early recognition by dental professionals is essential for prompt diagnosis and timely initiation of risk‐adapted surveillance strategies.

## 1. Introduction

Cowden syndrome (CS) is a rare hereditary disorder within the broader spectrum of PTEN hamartoma tumor syndromes (PHTSs), a group of conditions caused by germline pathogenic variants in the PTEN tumor‐suppressor gene [1]. Loss or dysfunction of PTEN results in dysregulation of the PI3K/AKT/mTOR signaling pathway, promoting abnormal tissue growth and contributing to hamartoma formation, alongside an increased predisposition to several malignancies [2]. CS follows an autosomal dominant inheritance pattern and exhibits high penetrance, although its clinical expression varies considerably among affected individuals [3].

Mucocutaneous lesions represent hallmark features of CS and are observed in the majority of adult patients. These include facial papules, acral keratoses, and characteristic oral papillomatous lesions that may involve multiple intraoral sites such as the gingiva, lips, and tongue. Patients often report the onset of these manifestations during childhood. Macrocephaly is another frequent finding and may support early diagnostic suspicion, particularly in younger individuals [1, 3]. The gastrointestinal and endocrine systems are also commonly affected in CS. The gastrointestinal and endocrine systems are also commonly affected in CS. Both the frequency and clinical presentation of manifestations differ markedly among patients, contributing to the overall phenotypic heterogeneity of the disorder [4]. As a result, individuals may exhibit only a limited subset of features or may develop additional manifestations progressively throughout life. This variability frequently complicates the diagnostic process, especially when typical mucocutaneous findings are subtle or not promptly recognized as syndromic indicators [5, 6].

Beyond benign or structural abnormalities, CS is associated with a substantially increased lifetime risk of several cancers, most notably breast, thyroid, endometrial, and renal malignancies [7]. These neoplasms may arise at younger ages and occur at rates higher than those observed in other hereditary cancer syndromes, underscoring the clinical consequences of delayed or missed diagnosis. Early identification of affected individuals is essential to ensure timely enrolment into dedicated surveillance programs aimed at cancer prevention and early detection [8].

Given the broad clinical spectrum and variability in disease expression, early diagnosis depends on the prompt recognition of key syndromic features in conjunction with detailed clinical history and genetic assessment. Genetic testing should be pursued when multiple organ systems exhibit suggestive patterns. Identification of pathogenic PTEN variants is required for diagnostic confirmation and enables the implementation of personalized, lifelong surveillance strategies that improve long‐term outcomes.

This report describes the case of a young woman presenting with extensive papillomatous intraoral lesions, referred to a tertiary referral oral medicine and oral surgery center, and illustrates the diagnostic pathway that led to an early recognition of CS.

## 2. Case Presentation

A 25‐year‐old woman was referred to a tertiary referral center for evaluation of multiple oral lesions that had progressively increased since childhood, representing the main reason for clinical assessment. The patient reported that the lesions first appeared during infancy and had gradually expanded in number and distribution over time. She denied bleeding, pain, or functional impairment.

Extraoral examination revealed macrocephaly (Figure 1), with an occipitofrontal circumference of 59 cm, exceeding the National Comprehensive Cancer Network (NCCN) threshold for adult women (≥ 58 cm).

**Figure 1:**
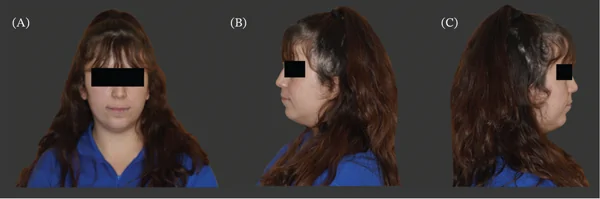
Extraoral examination. (A) Front view, (B) left profile, and (C) right profile.

Intraoral inspection showed extensive multifocal papillomatous lesions involving the upper and lower labial mucosa, the dorsum and lateral borders of the tongue, the hard palate, and the bilateral buccal mucosa (Figure 2). The lesions appeared as soft, sessile papules with a smooth to finely verrucous surface and showed a widespread, multifocal distribution and a long‐standing evolution, strongly suggesting an underlying systemic or hereditary condition rather than isolated reactive or infectious etiologies. The initial differential diagnosis included multifocal epithelial hyperplasia (Heck disease), HPV‐associated lesions, multiple squamous papillomas, reactive papillary hyperplasia, and other hamartomatous syndromes. However, the progressive long‐term course, diffuse oral involvement, and the coexistence of macrocephaly and thyroid structural lesions were considered more suggestive of an underlying syndromic disorder.

**Figure 2:**
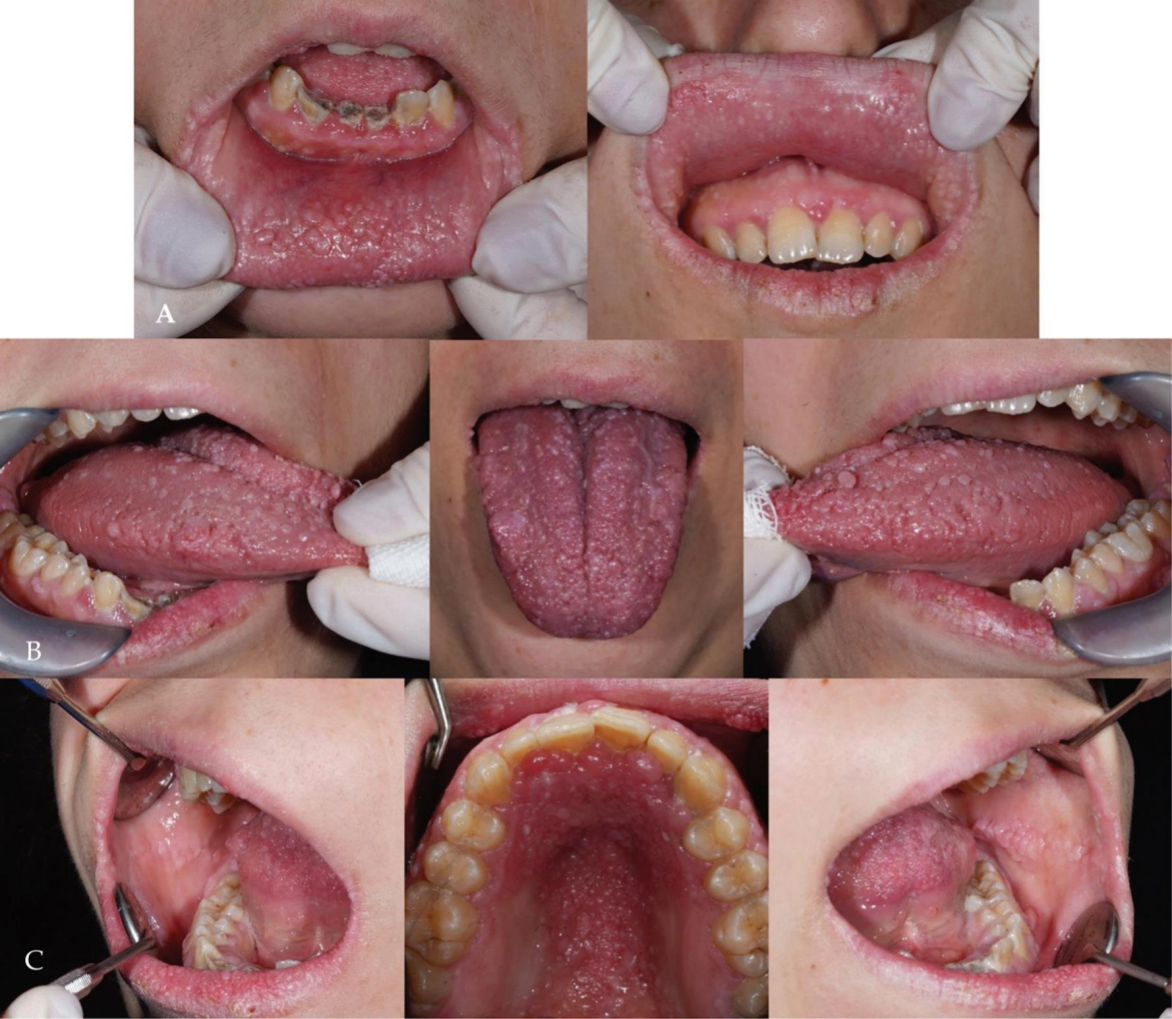
Intraoral examination. (A) Papillomatous lesions on the upper and lower labial mucosa. (B) Papillomatous lesions on the dorsum and lateral borders of the tongue. (C) Papillomatous lesions on the hard palate and buccal mucosa.

To characterize the lesions, an incisional biopsy was obtained from a representative papule on the lower lip.

Histopathological examination revealed a verrucous epithelial proliferation with mild undulating superficial parakeratosis, acanthosis, and elongated rete ridges extending into the underlying connective tissue. The epithelial component displayed mild cytological atypia consistent with low‐grade dysplasia, without architectural disorganization or evidence of invasive transformation (Figure 3). In view of the clinical and histological features, additional investigations were performed to further characterize the lesion and exclude alternative diagnoses: No viral cytopathic changes were identified, and both p16 immunostaining and HPV testing were negative, making an HPV‐driven lesion unlikely. Overall, these findings supported a nonviral proliferative process and, in the context of the patient′s clinical presentation and systemic findings, strengthened the suspicion of an underlying syndromic condition.

**Figure 3:**
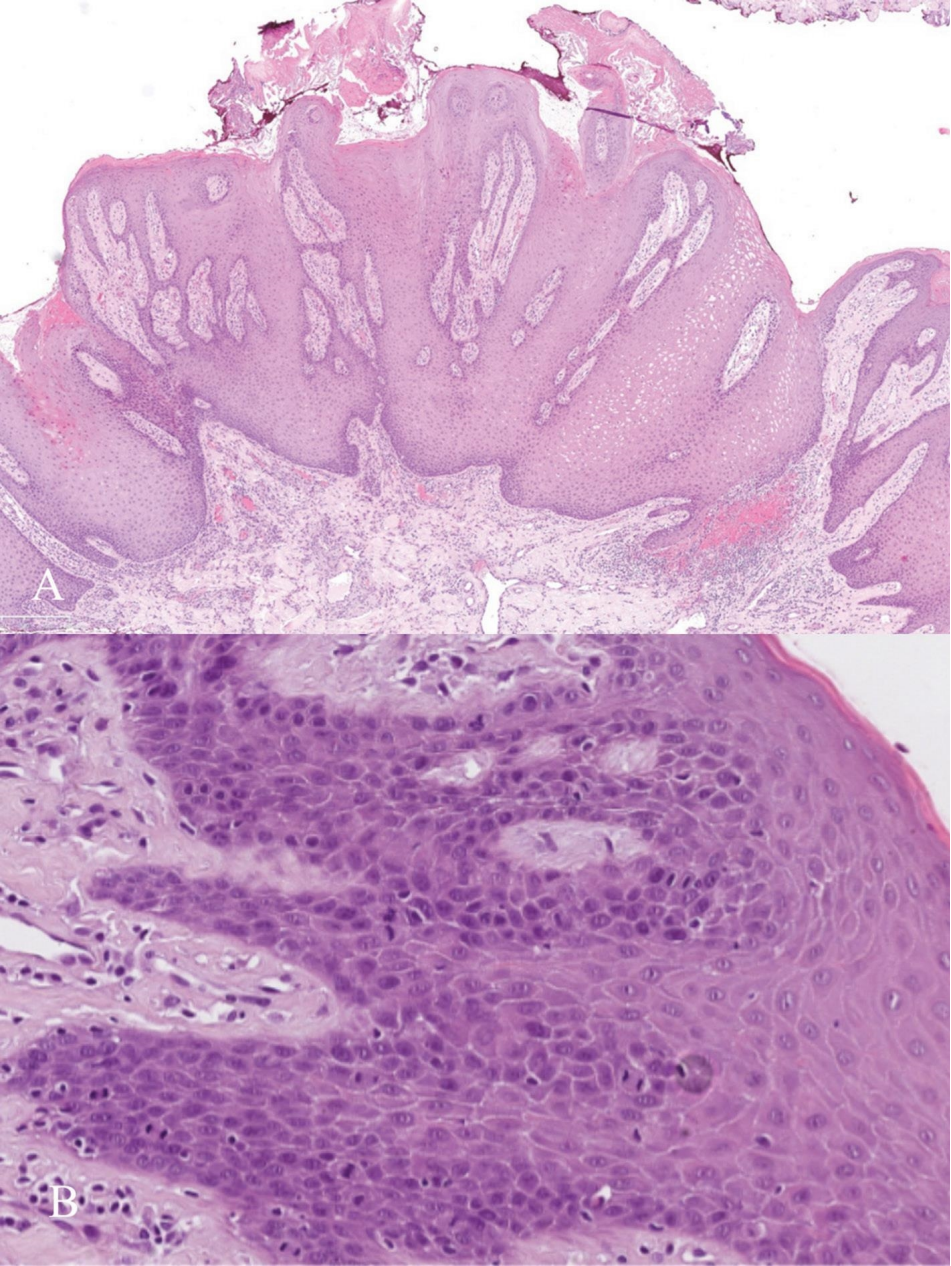
(A) Histologic analysis (5x) revealing a proliferative verrucous epithelial lesion displaying mildly undulating superficial parakeratosis, acanthosis, and markedly elongated, downward‐projecting rete ridges with associated low‐grade epithelial dysplasia. (B) Higher magnification (20x) demonstrating focal low‐grade epithelial dysplasia characterized by basal and parabasal hyperplasia, nuclear hyperchromatism, mild cellular and nuclear pleomorphism, an increased nuclear‐to‐cytoplasmic ratio, and mitotic figures predominantly confined to the lower third of the epithelium, whereas epithelial maturation is preserved in the superficial layers (hematoxylin and eosin stain).

In light of the histopathological findings and the unusual extent and chronicity of the oral papillomatosis, a broader clinical evaluation was undertaken. Review of the patient′s medical history revealed the presence of the following:

• Three benign thyroid nodules, diagnosed during adolescence, for which she had been receiving levothyroxine therapy since the age of 13;
• An 8‐mm sessile gastric polyp with a hyperemic and eroded surface identified during a previous gastroenterological assessment;
• No history of tobacco or alcohol use.

The coexistence of diffuse oral papillomatosis with onset in childhood, macrocephaly, benign thyroid nodules, and a documented gastric polyp raised a strong clinical suspicion of a PHTS, particularly CS. The concordant clinical, odontostomatological, and histopathological findings supported the indication for genetic testing.

Genetic evaluation was performed by whole‐exome sequencing on DNA extracted from a peripheral blood sample, followed by targeted analysis of genes associated with hamartomatous tumor syndromes. The analysis identified a heterozygous nonsense variant in PTEN, NM_000314.8:c.388C>T (p.Arg130Ter), introducing a premature stop codon and predicted to result in nonsense‐mediated mRNA decay with consequent loss of PTEN protein function. This variant is extremely rare in the general population (gnomAD 4.1: 0.0002%) and has been previously reported in association with CS, fulfilling criteria for classification as a pathogenic variant (Class V) according to ACMG/AMP guidelines.

The alignment between the clinical presentation, histopathological findings, systemic manifestations, and molecular results allowed a definitive diagnosis of CS. Following diagnostic confirmation, a multidisciplinary follow‐up plan was established, with referral to the appropriate specialties for comprehensive baseline assessment and implementation of a dedicated surveillance program.

Following diagnostic confirmation, a multidisciplinary follow‐up plan was established, with referral to the appropriate specialties for comprehensive baseline assessment and implementation of a dedicated surveillance program.

Regarding the oral lesions, given the presence of focal low‐grade epithelial dysplasia, a clinical follow‐up program at 3‐month intervals was established. At the time of writing, the lesions remained clinically stable, with no significant changes in morphology or extension. In the event of clinical modification or the appearance of suspicious features, repeat biopsy and further histopathological evaluation are planned.

As part of the multidisciplinary follow‐up, a thyroid ultrasound was performed which identified an increased size of one of the already known thyroid nodules and the presence of two enlarged lymph nodes. For this reason, the patient underwent endocrinological surveillance with a programmed needle biopsy of the thyroid nodules. In addition, considering the increased lifetime risk of breast cancer associated with PHTS, the patient was referred to a dedicated high‐risk breast cancer surveillance program.

Considering the autosomal dominant inheritance pattern of PHTS, family investigation was proposed following molecular confirmation, and genetic counseling was offered to first‐degree relatives. Clinical evaluation was performed on the patient′s parents and brother. The 15‐year‐old brother presented with a clinical picture characterized by focal epilepsy, likely secondary to a cerebrovascular anomaly, and a neurodevelopmental disorder, and his anthropometric parameters were as follows: head circumference (HC) 56.8 cm (90th centile), length 180 cm (91st centile), and weight 94 kg (90th centile). He was previously evaluated in another hospital where an in silico panel through WES for monogenic disorders associated with epilepsy (not including PTEN) was performed, resulting in a negative result. These clinical features and the co‐occurrence of the neurodevelopmental disorder raised suspicion for a PHTS‐related disorder (e.g., Bannayan–Riley–Ruvalcaba syndrome). Although these clinical findings prompted consideration of a possible PHTS‐related disorder, the brother′s HC at the 90th centile does not meet the criterion for macrocephaly. Furthermore, his previous epilepsy panel did not include PTEN; therefore, his PHTS status remains unconfirmed pending completion of the ongoing segregation analysis. The mother presented with isolated macrocephaly with an HC of 57 cm (> 97th centile), in the absence of any other signs or symptoms of PHTS. Moreover, the maternal family history was positive for bilateral breast cancer, hysterectomy in two of the mother′s aunts (sisters of her father) for unknown reasons, and one case of endometrial cancer in one of her cousins, raising suspicion for a possible *PTEN* variant segregation within the maternal lineage of the proband. Accordingly, with these findings, a segregation analysis of the identified *PTEN* variant in the brother and parents is currently ongoing at the time of writing.

Written informed consent was obtained from the patient for publication of clinical data and images.

The patient expressed relief at receiving a definitive diagnosis after years of clinical uncertainty and acknowledged the importance of identifying the underlying genetic condition. She reported good acceptance of the proposed multidisciplinary surveillance program and expressed reassurance that regular follow‐up would allow early detection and management of potential complications.

A summary of the main clinical milestones is reported below in accordance with CARE guidelines.

The clinical timeline is detailed as follows:

• Early childhood: onset of multifocal oral papillomatous lesions
• Adolescence: progressive increase in lesion number and extension
• Prediagnosis: detection of thyroid nodules and gastrointestinal polyps
• Diagnostic work‐up: oral biopsy and histopathological examination
• Diagnostic confirmation: identification of a germline PTEN pathogenic variant
• Postdiagnosis: initiation of family investigation and genetic counseling
• Follow‐up: oral lesions stable under 3‐month surveillance, thyroid nodule under endocrinological follow‐up, and enrolment in a high‐risk breast surveillance program.

## 3. Discussion

Severity of the phenotypic expression of CS may differ markedly even among individuals carrying the same pathogenic PTEN variant, complicating the identification of clear genotype–phenotype correlations. Moreover, the same PTEN pathogenic variant could be found in subjects presenting with different phenotypes in the PHTS spectrum (i.e., CS, Bannayan–Riley–Ruvalcaba, PTEN‐related Proteus syndrome, and Proteus‐like syndrome) even among individuals from the same family [2, 9–11]. Timing of occurrence of complications, especially PHTS‐related malignant neoplasms, varies too, thus imposing early surveillance in affected subjects, even in the presence of an apparently mild phenotype.

The PTEN variant identified in this study (c.388C>T; p.Arg130Ter) is a truncating pathogenic variant causing loss of PTEN protein function. Recent genotype–phenotype analyses suggest that truncating PTEN variants may be associated with a broader clinical burden and earlier disease expression compared with other variant classes; however, substantial phenotypic variability remains, even among individuals carrying identical variants, limiting the possibility of establishing a fully predictive genotype–phenotype correlation [12].

Given these heterogeneous clinical manifestations, standardized diagnostic criteria are fundamental for guiding clinical decision‐making. The International Cowden Consortium first developed a structured diagnostic framework [4], later revised by Pilarski et al. [8] and still regularly updated by the NCCN Guidelines v.3.2026 [13], which classifies clinical features into major and minor criteria (Table 1). These models have improved the identification of patients eligible for PTEN genetic testing, even in the absence of malignancy.

**Table 1:** Revised clinical diagnostic criteria for Cowden syndrome (NCCN v.3.2026).

**Major criteria**
1. Breast cancer
2. Endometrial cancer
3. Follicular carcinoma of the thyroid gland
4. Gastrointestinal hamartomas (including ganglioneuromas; excluding hyperplastic polyps; ≥ 3)
5. Adult‐onset Lhermitte–Duclos disease
6. Macrocephaly (> 97th percentile: ≥ 58 cm in women, ≥ 60 cm in men)
7. Macular pigmentation of the glans penis
8. Multiple mucocutaneous lesions (see subcriteria)
**Minor criteria**
1. Autism spectrum disorder
2. Colorectal cancer
3. Esophageal glycogenic acanthosis (≥ 3)
4. Lipomas (≥ 3)
5. Intellectual disability (IQ ≤ 75)
6. Renal cell carcinoma
7. Testicular lipomatosis
8. Thyroid cancer (papillary carcinoma or follicular variant)
9. Thyroid structural lesions (adenoma, adenomatous goiter, etc.)
10. Vascular anomalies (e.g., developmental venous anomalies)
**Diagnostic criteria for an individual**
An individual is diagnosed with CS/PHTS if *any* of the following is met
• Three or more major criteria, one of which must be macrocephaly, adult‐onset Lhermitte–Duclos disease, or gastrointestinal hamartomas
• Two or more major criteria *and* three or more minor criteria
**Diagnostic criteria for family members**
A family member is diagnosed with CS/PHTS if *any* of the following is met
• Two or more major criteria
• One major criterion and at least two minor criteria
• At least three minor criteria

Our case presented with diffuse oral papillomatosis, a pathognomonic mucocutaneous finding considered a major criterion according to the NCCN Guidelines v.3.2026 [13], together with macrocephaly, representing a second major criterion, and thyroid nodules, accounting for one minor criterion. Although gastrointestinal polyps were also reported, these were not considered in the diagnostic work‐up due to the lack of histopathological confirmation of their hamartomatous nature. Therefore, the patient fulfilled two major and one minor criterion, which were insufficient to establish a clinical diagnosis of CS. This presentation mirrors patterns described in several case series documenting early, predominantly mucocutaneous manifestations of CS [10, 14, 15]. In this context, molecular analysis proved decisive for diagnostic confirmation, underlining the importance of early referral for genetic evaluation in patients with suggestive but incomplete phenotypic presentations.

Oral manifestations play an important role among diagnostic indicators. Several studies, including those by Hammerschmidt et al. [16], Flores et al. [17], and Celentano et al. [18], identify oral papillomatous lesions as some of the earliest and most prevalent signs of CS, occurring in up to 80%–100% of adults with confirmed pathogenic PTEN variants. These lesions typically present as multiple small papules affecting the lips, tongue, gingiva, or buccal mucosa, with onset often traceable to childhood. Importantly, oral lesions may appear long before systemic manifestations, making them valuable early markers of an underlying genetic disorder. Flores et al. [17] emphasized the central role of dentists in early detection, noting that oral findings were among the primary reasons for referral to specialists in their cohort. More recent studies have reinforced this concept, showing that oral manifestations may represent some of the earliest and most clinically useful signs for recognizing PHTS [19, 20].

Making a comparison with previously reported cases further highlights the clinical relevance of the present report. In the clinical case described by Seol et al. [21], oral papillomatous lesions were recognized only at 53 years of age and developed shortly before diagnosis, in a patient who had already developed breast cancer and multinodular goiter. Similarly, Marshall et al. [22] reported a 58‐year‐old patient in whom oral lesions led to diagnosis, but in the presence of additional extraoral mucocutaneous findings. More recently, Yuan et al. [23] described a 41‐year‐old patient with rare oral manifestations associated with CS, and Tavares et al. [24] reported a late‐diagnosed case requiring oral rehabilitation: These reports emphasize the pivotal role of dental professionals not only in the early recognition of PTEN‐related disorders but also in improving oral function and quality of life through rehabilitative treatment.

In contrast, our patient presented with progressive multifocal oral papillomatosis since early childhood, without characteristic dermatological signs such as trichilemmomas or acral keratoses, as reported in previously published cases [16, 17]. Therefore, the oral phenotype represented the main clinical clue prompting genetic investigation. This aspect is particularly relevant because recent studies have emphasized that oral features may be among the earliest and most clinically useful signs for recognizing PHTS [19, 20]. This case underscores the importance of recognizing persistent multifocal oral papillomatous lesions as potential early indicators of PTEN‐related disorders, warranting prompt genetic evaluation and surveillance [25].

Despite their diagnostic relevance, oral lesions may be misinterpreted as viral warts, multifocal epithelial hyperplasia, HPV‐associated lesions, reactive papillary hyperplasia, or other hamartomatous syndromes, which highlights the essential role of dental professionals who are frequently the first clinicians to observe these abnormalities. Hammerschmidt et al. [16] demonstrated that oral papules in CS may clinically and histologically resemble viral warts, showing papillomatosis, acanthosis, and koilocytosis. Webber et al. [26] reported that prompt recognition of unusual mucosal changes in dental settings can significantly reduce diagnostic delays by facilitating appropriate referrals. Similarly, Glavina et al. [27] described cases in which gingival papillomatosis was the key finding leading to CS diagnosis, underscoring the need for interdisciplinary awareness. Sousa‐Neto et al. [28] further highlighted the diagnostic value of oral manifestations in clinically heterogeneous presentations. Collectively, these observations demonstrate that oral medicine specialists, periodontists, and maxillofacial clinicians play a central role in the early detection of PHTS.

Histopathological findings in the present case add a further element of interest. Previous reports have mainly described Cowden‐associated oral lesions as benign papillary or fibroepithelial proliferations characterized by acanthosis, hyperkeratosis, elongated rete ridges, and variable inflammatory infiltrate, with dysplastic changes being only occasionally mentioned or not specifically discussed [23, 28]. In our case, focal low‐grade epithelial dysplasia was observed in association with diffuse basal layer activation, whereas the overall epithelial architecture remained preserved and no evidence of invasive transformation was identified. The clinical significance of this finding remains to be clarified. The observed focal low‐grade dysplasia may reflect the prolonged clinical evolution and persistent epithelial proliferation of the lesions, potentially broadening the histopathological spectrum of Cowden‐associated oral manifestations.

However, a rare report of oral squamous cell carcinoma in patients with CS suggests that epithelial instability in PTEN‐related disorders may deserve further attention [29]. Although current evidence is limited, the presence of dysplastic changes in our case supports careful histopathological interpretation and periodic oral follow‐up, particularly in lesions showing persistence or clinical progression.

Once the diagnosis of CS has been confirmed, cancer surveillance is essential due to the high cumulative risk of neoplasms over the course of a lifetime, up to 85% for breast cancer, 35% for thyroid cancer, 28% for endometrial cancer, and 34% for kidney cancer, as reported by Tan et al. [7].

The PHTS surveillance guidelines and NCCN recommendations indicate a personalized, multidisciplinary screening program focused on the organs most at risk. These include the following:

• Annual breast MRI from Age 30, with the addition of mammography between Ages 30 and 35;
• Annual thyroid ultrasound from childhood;
• Colonoscopy every 5 years from Age 35;
• Renal imaging (ultrasound or MRI) every 1–2 years from Age 40;
• Periodic gynecological evaluation from Ages 30 to 35, with education on early symptoms (e.g., abnormal uterine bleeding) [7, 20, 21].

These coordinated surveillance strategies reflect a growing international consensus that early and proactive monitoring significantly improves outcomes in individuals with PHTS. As reported by Dragoo et al. [30], adherence to structured screening protocols increases the likelihood of detecting malignancies at earlier and more treatable stages, reinforcing the necessity of integrating genetics, oncology, and primary care in long‐term management.

The importance of a multidisciplinary approach is widely recognized in the care of individuals with PTEN‐related disorders. Because multiple organ systems may be affected across the lifespan, coordinated care among endocrinologists, dermatologists, gastroenterologists, gynecologists, oncologists, nephrologists, and clinical geneticists is essential [1, 2, 9].

Given the substantial intrafamilial variability in clinical expression and incomplete penetrance of PHTS, cascade testing in relatives is of utmost importance even in the absence of strong clinical suspicion, as highlighted by the extensive literature and clinical practice recommendations [1, 3]. Identifying asymptomatic carriers early enables timely implementation of surveillance and preventive measures, which may reduce cancer‐related morbidity and mortality [1, 7, 9].

## 4. Conclusion

This case highlights the pivotal role of dental professionals in the early diagnosis of CS and underscores the importance of considering PHTS when persistent mucocutaneous lesions coexist with visceral findings, particularly in young individuals. The combination of clinical assessment, multidisciplinary evaluation, and molecular confirmation enables timely enrolment in evidence‐based surveillance programs, ultimately improving prognosis and quality of life for affected patients.

## Author Contributions

Conceptualization: Gian Marco Podda, Lucia Borghetti, and Umberto Romeo; data curation: Lucia Borghetti and Federica Rocchetti; formal analysis: Lucia Borghetti, Gian Marco Podda, and Cira Rosaria Tiziana Di Gioia; investigation: Gian Marco Podda, Lucia Borghetti, and Federica Rocchetti; methodology: Gian Marco Podda, Lucia Borghetti, and Niccolò Di Giosaffatte; project administration: Umberto Romeo; resources: Niccolò Di Giosaffatte, Irene Bottillo, Paola Grammatico, and Cira Rosaria Tiziana Di Gioia; supervision: Umberto Romeo; validation: Niccolò Di Giosaffatte, Irene Bottillo, Paola Grammatico, and Cira Rosaria Tiziana Di Gioia; visualization: Lucia Borghetti; writing—original draft: Lucia Borghetti, Gian Marco Podda, and Niccolò Di Giosaffatte; writing—review and editing: Lucia Borghetti, Gian Marco Podda, and Niccolò Di Giosaffatte.

## Funding

No funding was received for this manuscript. Open access publishing facilitated by Universita degli Studi di Roma La Sapienza, as part of the Wiley ‐ CRUI‐CARE agreement.

## Disclosure

All authors have read and approved the final version of the manuscript. Lucia Borghetti had full access to all of the data in this study and takes complete responsibility for the integrity of the data and the accuracy of the data analysis.

## Ethics Statement

According to institutional policy, formal ethical approval was not required for the publication of a single anonymized case report. Written informed consent was obtained from the patient for the publication of clinical details and images. The study was conducted in accordance with the principles of the Declaration of Helsinki.

## Consent

Written informed consent was obtained from the patient for the publication of this case report and the accompanying clinical images.

## Conflicts of Interest

The authors declare no conflicts of interest.

## Data Availability

The data supporting the findings of this case report are available from the corresponding author upon reasonable request.
